# Home-Based Pilot Intervention to Improve Quality of Life and Related Outcomes among Unpaid Caregivers and Their Care-Recipients with Visual Impairments

**DOI:** 10.3390/ijerph20053867

**Published:** 2023-02-22

**Authors:** Afeez Abiola Hazzan, Pamela Beach, Lauren J. Lieberman, Cassidy Regan

**Affiliations:** 1 Department of Healthcare Studies, State University of New York Brockport, State University of New York, 350 New Campus Drive, Brockport, New York, NY 14420, USA; 2 Department of Kinesiology, Sport Studies & Physical Education (KSSPE), State University of New York Brockport, State University of New York, 350 New Campus Drive, Brockport, New York, NY 14420, USA

**Keywords:** blindness, low vision, caregiving, burden, physical activity, mental health, wellness, stress, caregiving, interventions

## Abstract

The increasing lifespan of the United States population has led to a rise in the prevalence of age-related chronic diseases, increasing the need for unpaid caregivers. Currently, little research is known about this specific population beyond the limited formal training unpaid caregivers receive on the caregiving process. Acquiring visual impairments (VI) later in life places a large emotional toll on both the loved one and their caregivers. The objectives of this pilot study were: (1) to implement a multimodal intervention targeted towards improving the quality of life of unpaid caregivers and their care recipients with visual impairments; (2) to evaluate the efficacy of the multimodal intervention in improving the quality of life of unpaid caregivers and their care recipients with visual impairments. A 10-week virtual intervention (e.g., tai chi, yoga, music) was implemented with 12 caregivers and 8 older adults with VI. The targeted outcomes of interest included: QoL, health, stress, burden, problem-solving, and barriers. In addition to surveys to inform the choice of the intervention, focus group interviews were conducted to obtain participants’ perspectives on the effectiveness of the intervention. Results revealed many positive outcomes in the quality of life and well-being of the participants following the 10-week intervention. Overall, these results represent a promising program for unpaid caregivers of older adults with VI.

## 1. Introduction

The population of older adults in the United States has been progressively increasing. In 2019, the population age 65+ was 54.1 million–30 million women and 24.1 million men [1]. An increasing lifespan leads to a rise in the prevalence of chronic diseases and impairments associated with the aging process [1,2]. Not surprisingly, the need for caregivers is at a high; the National Study of Caregiving (NCOS) estimated that there were over 22 million informal caregivers in the United States in 2014, with the majority of that number being from older adults between 65 and 75 years of age [2]. Among all caregivers of older adults in the United States in 2011, 13% cared for individuals with only visual impairments, and an additional 10% cared for individuals with both visual impairments and dementia [3], revealing a prevalence of 23% of all caregivers for older adults with visual impairments. With a demographic shift towards increasing age, the need for caregivers of older adults with visual impairments will continue to rise.

Most unpaid caregivers are often placed in their role without having any formal training or knowledge on the caregiving process, but are still expected to function as unrecognized extensions of healthcare for their loved ones. Vision loss is particularly difficult to care for due to the large emotional toll for the patient and the challenge of assisting with tasks which were formerly easy for the patient to perform [4]. The Center for Disease Control reports that vision loss has been linked to feelings of worry, anxiety, loneliness, fear, and social isolation in the people experiencing it [4]. This is especially the case when the vision loss is transient due to the aging process and from common age-related diseases such as macular degeneration, glaucoma and cataracts [5]. Transient vision loss in adults is different from adults with congenital vision loss because losing your vision late in life is sudden and disrupts many aspects of life. Transient vision loss reduces an individual’s capacity to drive a car, read, watch television, and keep personal accounts such as private banking information [6]. It also often isolates older adults and keeps them from being able to leave their home to visit or contact friends and family. Caregiving responsibilities specific for older adults with visual impairments include assisting with activities of daily living such as reading, transportation, financial management and personal care, as well as complying with the medical needs of the patient such as medication management, and often assisting with therapeutic rehabilitation directions [7]. In the study by Varadaraj et al. (2020), caregivers of individuals with both dementia and VI spent 1.7 times, and caregivers of individuals with only dementia or VI spent 1.3 times as many hours providing care than caregivers of those without either disability [3]. Additionally, caregivers of a loved one with both dementia and VI had 3.2 times as many valued activities affected while caregivers of only dementia and only VI reported 1.9 times, and 1.3 times more activities affected, respectively. The combination of increased time caregiving and decreased opportunities to engage in valued activities takes a large emotional toll on many aspects of the caregivers’ life.

The negative effect of unpaid caregiving is so well researched that it has been termed caregiving burden [8]. Caregiving burden describes the negative psychological, behavioral, and physiological impact of caring for someone who is ill or has functional impairments. The phenomenon of caregiving burden that can cause psychological distress and depression has also been linked to causing negative hormonal changes, an increase in susceptibility to infection and disease, and a disruption to healthy habits such as getting enough sleep and eating well [9]. Since caregivers are placed in high stress situations and are at a higher risk of neglecting their own needs and leisure activities, it is important to support these individuals to lessen their burden and improve their quality of life.

Currently, little research has been conducted on the specific population of unpaid caregivers for older adults with visual impairments [10]. Since caring for older adults with visual impairments comes with specific responsibilities and difficulty, further knowledge needs to be gained on how the informal caregivers for this population need to be supported. However, the literature consistently shows a moderate relationship between the level of patient disability and the psychological distress of the caregiver [11].

The purpose of this study was to pilot an intervention to improve the quality of life and well-being of unpaid caregivers and their care recipients with visual impairments. The objectives of this study were: (1) to implement a multimodal intervention targeted towards improving the quality of life of unpaid caregivers and their care recipients with visual impairments; (2) to evaluate the efficacy of the multimodal intervention in improving the quality of life of unpaid caregivers and their care recipients with visual impairment. The outcomes of interest that were targeted as part of this study included: quality of life, health, stress, burden, problem-solving, and barriers among unpaid caregivers as well as their loved ones. The current report focuses on the implementation and preliminary results of a virtual home intervention for unpaid caregivers and their care recipients with visual impairments.

## 2. Materials and Methods

### 2.1. Participants

Participants were recruited through social media, personal invitations, and caregiver-serving organizations throughout the United States. Inclusion criteria included unpaid caregivers of adults with visual impairments over the age of 30. Care-recipients must have been diagnosed with a visual impairment by a medical practitioner as reported by the caregiver. Additionally, participants needed to have internet access and basic computer literacy. Recruitment took place between May to July 2021. Refer to Table 1 for the descriptive statistics of the participants. Approval from the local researcher’s institutional review board was obtained prior to recruiting participants.

For this pilot study, a 10-week virtual intervention was implemented with 12 caregivers and 8 older adults with visual impairments, for a total of 20 participants. The eight older adults with visual impairments participated with their caregivers, while the remaining four caregivers participated on their own. Caregivers completed a pre-study survey to inform the choice of the intervention, as well as a post-study survey. The pre-study survey was conducted to obtain demographic information about the caregiver and care recipient. The pre-study survey was also used to collect the caregivers’ choice of intervention to be implemented. The post-study survey was used to obtain feedback from the caregivers about the intervention as well as any suggestions for improvement. In addition, focus group interviews were conducted to obtain participants’ perspectives on the effectiveness of the intervention. The focus group participants included both caregivers and care recipients.

### 2.2. Intervention Procedures

The intervention was held for one hour per week for a total of 10 consecutive weeks. Table 2 provides the list of all activities included in the intervention, as well as the facilitators, and a brief description of each intervention. 

The intervention was informed by the responses that participants provided in the pre study survey that preceded the intervention period. Both caregivers and care recipients participated at the same time. Additionally, each presentation or workshop was tailored to meet the needs of the caregiver and their care recipients by making it as descriptive as possible. The choice of the weekly workshops/presentation was suggested by the caregivers who completed the pre-study survey. These participants also suggested the weekly meeting time to allow for maximum participation. Topics covered in the intervention included tai chi, yoga, meditation, dancing, music and singing, activities of daily living such as packing a suitcase for travel, cooking, gardening, coin collecting, and birding. The individuals providing the intervention were college professors with expertise in working with individuals with visual impairments, tai chi by a local expert, activities of daily living by a woman who is blind, as well as several workshops and demonstrations by members of the study team. Further, the hobbies of gardening, coin collecting, and birding were provided by one of the participants who also acted as a co-facilitator for some of the weekly sessions.

To counteract the impact of the ongoing COVID-19 pandemic, the intervention components were delivered virtually over Zoom. Each session was recorded and access was provided to all participants to watch the sessions through Google Drive afterwards. Participants were asked to complete a weekly time log, reporting the activities they engaged in, including watching the recording. The efficacy/effectiveness of the intervention components was evaluated by measuring and comparing important outcomes such as quality of life before (i.e., “pre”) and after (i.e., “post”) the 10-week intervention period. A pre–post study design is ideal for measuring the occurrence of an outcome before and then after a particular intervention is implemented [12]. For the current study, a single arm pre–post design was used. In addition to the questionnaires, participants took part in a focus group interview at the end of the 10-week intervention period. Two focus groups were completed, with a total of five to seven participants in each session. Each focus group had both caregivers and care-recipients. The focus group interviews were led by three of the authors using questions that had been piloted.

### 2.3. Measures

For this study, caregivers completed a series of questionnaires before (pre-survey) and after (post-survey) the intervention period. These questionnaires include: the Satisfaction with Life Scale, Living Arrangement and Indicators of Social Interaction survey, Caregiver quality of life (EQ-5D), the Perceived Change Index (PCI), and the Geriatric Depression Scale (GDS). These surveys were selected because they provide information about quality of life and related outcomes at the time they were administered.

The Satisfaction with Life Scale [13] is a 5-item questionnaire which includes responses from 1 to 7 on a Likert scale. The Satisfaction with Life Scale has favorable psychometric properties with 0.73 correlation with other measures of subjective well-being. The item-total correlations for the five items were: 0.81, 0.63, 0.61, 0.75, and 0.66, showing a good internal consistency with the scale. Further, the two-month test–retest reliability was 0.82 [13]. Participants also completed the Living Arrangement and Indicators of Social Interaction survey which is a 9-item measure examining the amount and types of social interactions of individuals [14,15].

In addition to the above, the study participants completed two surveys developed specifically for caregivers, including the Caregiver quality of life (EQ-5D) and the Perceived Change Index [16,17]. The EQ-5D is broken down into 5 topical areas including mobility, self-care, usual activities, pain/discomfort, and anxiety/depression. The EQ-5D has been shown to have excellent psychometric properties including test–retest reliability with a scale agreement of 0.7 and convergent validity of 0.76 [16]. The PCI examines the perceived change in the caregiver’s well-being. Psychometric analyses of the PCI showed that it is valid and internally consistent with Cronbach’s alpha of 0.90 [17].

Further, the Geriatric Depression Scale (GDS) was used to measure depression and mental health [18]. The GDS has been tested and extensively used in older adults. The GDS has been shown to be a valid and reliable tool. Both the long form and short form of the questionnaire were able to differentiate depressed from non-depressed adults with a high correlation of 0.84 (*p* < 0.001) [19]. In addition, the GDS Long Form is a brief, 30-item questionnaire in which participants are asked to respond by answering yes or no in reference to how they felt over the past week. A Short Form GDS consisting of 15 questions was developed in 1986. Questions from the Long Form GDS which had the highest correlation with depressive symptoms in validation studies were selected for the short version. Of the 15 items, 10 indicated the presence of depression when answered positively, while the rest (question numbers 1, 5, 7, 11, 13) indicated depression when answered negatively. Scores of 0–4 are considered normal, depending on age, education, and complaints; 5–8 indicate mild depression; 9–11 indicate moderate depression; and 12–15 indicate severe depression. The Short Form is more easily used by physically ill and mild.

The qualitative focus group interview questions were developed by three of the authors who are very well versed in qualitative research. Face and content validity of the scripts were established with reviews by two caregiver researchers (both researchers on this paper), one caregiver (not on this paper), and one researcher in visual impairment (researcher on this paper). The questions were then revised two times with revisions made to the wording and the order of questions until consensus was reached. The process of analyzing questions followed Connell et al.’s (2018) [20] item analysis for the quality-of-life measures. The quality measures checks for item relevance, ease of response, item ambiguity, distressing or sensitive items, and judgement items. This instrument was not pilot tested before use.

### 2.4. Data Analyses

Quantitative data were collected using Qualtrics Web Survey Tool. The data consisted of demographic data, caregiving context, as well as the five questionnaires. Statistical analyses of the quantitative data collected from the surveys were completed using IBM SPSS Statistics (version 27). Descriptive statistics were utilized to profile the study participants and elucidate their caregiving context. Summary statistics were calculated for the five questionnaires to provide an overview of the sample data collected.

Two focus groups were held with five and seven participants excluding the facilitators. Data from the focus group interviews were transcribed verbatim, and the accuracy verified by the authors. Transcripts from the focus group interviews were analyzed using qualitative description methodology [21]. Three authors independently read the focus group interview transcripts to achieve robust understanding of the data. The authors performed independent coding and then met to compare codes, including sorting the codes into emergent themes that represent the key findings from the focus group interviews. The researchers met three times to review their emergent themes and determine subthemes and associated quotes. Disagreements were discussed for each theme, mainly the wording used for the theme and the placement of the subtheme. Disagreements were discussed until a consensus was reached during the third meeting [21].

## 3. Results

### 3.1. Program Implementation

Although the small sample size precludes inferential statistics, descriptive analysis of key outcomes from the questionnaires are presented to show how they changed from pre- to post-intervention. Table 1 provides demographic information about the participants, including the caregivers and older adults with visual impairments. The majority (66.7%) of the caregivers were female, and the median age was 56.5 years. Further, the majority of the caregivers were white (58.3%), and a third (33.3%) were African American. Additionally, the majority (75%) of caregivers spent more than 8 h per day providing care for the care recipient, and the majority (58.3%) live with the care recipient. In addition, the median age for the care recipient was 71 years, with a range of 40 to 95 years. The majority of the care recipients were female (58.3%), with an equal number of white (41.7%) and African American (41.7%) people. The most common types of visual impairment that the care recipients exhibit are far sightedness/short sightedness requiring the use of corrective lenses (25%), total blindness in one or both eyes (16.6%), macular degeneration (16.6%), and color blindness (16.6%). Other types of visual impairments include retinitis pigmentosa and severe blurred vision.

#### Intervention and Workshops

Both unpaid caregivers and older adults with visual impairments reported that the 10-week virtual intervention and the accompanying workshops were informative and helped to improve their quality of life. Briefly, a variety of workshops were presented as part of the intervention, with paid professionals leading most of them. All workshops were conducted in a manner to be accessible to both the caregiver and the care recipients by including additional verbal instructions to detail any visuals. For example, during yoga or tai chi, additional verbal cues were provided and visual cues such as ‘look at the position of my hands’ were avoided. Several of the activities throughout the intervention were led by a participant who developed a strong interest in the study. The activities conducted during each workshop were chosen based upon the interest of the group. Members of the research team also led activities in areas that were related to their fields of expertise.

### 3.2. Program Efficacy

#### Summary of Results from the EQ-5D, PCI, GDS, Satisfaction with Life Scale, and Living Arrangement and Indicators of Social Interaction

Results from the pre- and post-surveys were compared for the five questionnaires completed by participants. Although values for some measures were higher at the conclusion of the intervention, statistical tests were not possible due to small sample size. For example, the findings showed that 21.7% more caregivers reported that they were satisfied with their life at the post intervention level compared to the pre intervention level. The other key outcomes that were higher at the conclusion of the intervention include: a feeling of helplessness (23.3%), if the feeling of being overwhelmed has improved a lot (11.7%), if the ability to understand care recipient’s behavior has improved a lot (10.0%), and having no problems with mobility (16.4%). However, a few other variables remained fairly constant or worsened slightly when comparing the pre- and post-intervention results. These variables include: feeling full of energy (−10.0%), if the ability to manage day-to-day caregiving has improved a lot (1.7%), having no pain/discomfort (−3.3%), and anxiety or depression (+25.0%). However, the inability to conduct statistical analysis due to a small sample size precludes meaningful inferences from these data.

### 3.3. Themes from Focus Group Interviews

Five major themes were discovered including: (1) time for self, (2) barriers, (3) learning opportunities, (4) quality of life, and (5) social support. The themes and their associated sub-themes are described below:

#### 3.3.1. Time for Self

The caregivers suggested that this intervention study allowed them the time to focus on their own needs. As sub-themes, the caregivers asserted that the intervention provided them an *escape* from their regular duties as caregivers, while also allowing them the opportunity to practice *self-care*. These are illustrated in the quotes below:

*“One thing you probably made me do is actually take some time to participate in this which is actually taking time for myself, which I would not otherwise have done if I hadn’t been invited.”*.

(Caregiver, female, 64 years old)

*“I tried it (activities) a couple times to do it with my wife. It was harder. I decided for my own self-care, it would make me happier just to try to do it without her as a lone ranger.”*.

(Caregiver, male, 67 years old)

*“It’s helping me to relax my mind and brain when I’m stressed out.”*.

(Caregiver, male, 35 years old)

#### 3.3.2. Barriers

Although the intervention components were helpful in alleviating the challenges of caregiving, many caregivers identified barriers to their participation in the study. These barriers included: technology, time, and opportunity costs of participating in the program. Technology was a barrier because some of the participants were not very comfortable with Zoom and some of the other software used in this study. Additionally, finding time from their busy schedule to attend the weekly intervention was challenging. Further, participants may have lost out on the benefits that are potentially accruable from participating in other competing activities, including spending alone time with their partner. Therefore, successful navigation of these barriers was important to maximizing the benefits of the intervention. The following quotes support this theme:

*One (barrier) was just the timing. Also, I struggled with the technology. I happen to have a computer explode on me at the time, certain headphones didn’t work, learning the technology of Zoom… (Care-recipient, female, 49 years old)“Sometimes, I was slow on clicking out of the video, and I would be in a video that I entered through Google Drive, but then it would come up and the next thing would be from YouTube.”*.

(Caregiver, male, 67 years old)

#### 3.3.3. Learning Opportunities

The intervention provided caregivers with opportunities to improve their quality of life and ability to manage the challenges of caring for an older adult with visual impairment. Additionally, participants planned to continue using the lessons learned from the intervention beyond the duration of the study. The following quotes illustrate this theme:

*…I think the meditation exercises, the singalong exercises with the group was definitely helpful… even learning some of the Tai Chi examples…We’re definitely going to try to implement that more into our routine…*.

(Caregiver, female)

*“Well, the weekly meeting, as you know, gave me some new ideas to practice or incorporate into my daily life. During my free time, I could bring back the tai chi, maybe on a Saturday just for practice.”*.

(Caregiver, male, 32 years old)

#### 3.3.4. Quality of Life

The caregivers perceived improvement in their quality of life as a result of participating in the study. Additionally, caregivers expressed the expectation that the improvement will continue beyond the study as they implemented various activities from the study into their daily lives. This theme and the associated sub-theme of continuity are illustrated by the quotes below:

*“I have a better quality of life. I don’t feel bored throughout the week…I come to this group, I have a good time, learn something new… So, it’s been great for me.”*.

(Caregiver, male, 32 years old)

*“My quality of life has really improved the past few weeks. You know, I’ve been happy lately. I started playing my chess game on pony. It’s fun. It’s cool. I’m less busy.”*.

(Caregiver, male, 35 years old)

*“I feel like all of these types of opportunities make me improve my quality of life.”*.

(Care-recipient, female, 49 years old)

#### 3.3.5. Social Support

Caregivers asserted that this study provided them with much needed social support, including a sense of community. This proved to be instrumental in caring for their loved one with a visual impairment, particularly during the pandemic. The following quotes highlight this theme:

*…“Being in community with other people who have the same or similar challenges is really beneficial. It doesn’t matter to me, whether they’re far away, twice my age… I just think there’s a lot to be gained…it helps my quality of life.”*

(Care-recipient, female, 49 years old)

*“To see you and other human beings and the smiling faces. The body language goes a long way. It really did make me feel like I wasn’t as isolated as I had been for the previous 16 months. Thank you all for brightening.”*

(Caregiver, male, 67 years old)

*“I’m with people who understand me better, so I share my pain and joy and maybe a little bit of experience.”*

(Caregiver, male, 32 years old)

## 4. Discussion

In this pilot study, 12 caregivers of loved ones with visual impairments and 8 care recipients engaged in a 10-week virtual intervention to improve their quality of life, as well as teach a variety of accessible recreational activities. It has been established that caregivers who care for an older loved one with a visual impairment spend up to three times more time helping their loved one than caregivers of loved ones without a visual impairment [3,11]. In this study, most of the caregivers were providing at least eight hours of unpaid caregiving to their loved ones every day, many of whom lived with their loved one, similar to the findings of Varadaraj et al. (2020) [3]. This additional time caregiving leaves limited time for preferred or valued activities by the caregiver and necessitates intervention components that will help the caregiver engage in valued activities while still caring for their loved one.

While recruitment was challenging, all of the participants who began the study not only completed the study but participated with high attendance, indicating the feasibility of this pilot intervention for a larger sample size. Perhaps of particular interest, one participant became so invested that he led several of the activity sessions throughout the intervention.

The activities were all chosen by the participants to allow more autonomy and increase interest. Activities such as cooking, tai chi, meditation, gardening, and packing for a trip are lessons that have the ability not only to reduce stress, but also to teach the participants how to navigate their lives while living with a visual impairment. As previously discussed, most unpaid caregivers have no background in supporting a person with a visual impairment, let alone additional disabilities such as dementia, Alzheimer’s, or a stroke [7]. These lessons not only showed how to engage in these enjoyable recreational activities, and independent living skill activities, but they also embedded how to modify them for their loved one with a visual impairment. These life lessons together helped the participants navigate the unchartered territory for caring for their loved one with a visual impairment giving the caregivers tools to use in their free time to relax, de-stress, and have some control over their lives. This may be one reason why several areas of life satisfaction improved. The fact that some areas stayed the same or even decreased could have been due to the fact that the caregivers saw how hard it may be to engage their loved one in some of these activities, or seeing the need for more help in order to engage them in each of these may also be the case.

Research has shown that these caregivers have lower levels of quality of life and are more likely to be stressed and anxious [9,10,11]. The participants in this study also revealed high levels of stress and anxiety, in addition to a lower quality of life in their EQ-5D and GDS scores. After the intervention, it was seen that anxiety, life satisfaction, and feeling overwhelmed improved. Other areas such as pain and discomfort, mobility, and feeling overwhelmed also went down slightly. Qualitative results from the focus groups further illuminated the benefits of a weekly meeting with other caregivers of individuals with visual impairments, in particular time for self, learning opportunities, quality of life, and social support. While participants expressed that the technology was a particular challenge, the opportunity to engage with other caregivers and focus on themselves for one hour per week was worth the challenge. Caregiving can be quite isolating, and the sense of community that was developed by this intervention can be highly beneficial to improving quality of life and well-being [22,23].

There were several limitations in this study, most notably the small sample size. Future research should extend this study to a larger sample size to further examine the effectiveness of a virtual home intervention upon improving the quality of life of unpaid caregivers of older adults with visual impairments. Larger sample sizes would also allow for the opportunity to separate caregiver and care recipient experiences. In addition, the qualitative results could have been strengthened through pilot testing of the focus group questions. Technology was a challenge for participants and may have limited recruitment due to limited internet access and computer literacy. An unexpected outcome of the study was a participant who participated as a facilitator in several workshops with short informational sessions on topics of interest. This participant acting as a participant with a minor role as a co-facilitator may have added possible bias. While this study was not without limitations, the results of this pilot study are encouraging. This intervention provided the participants with a form of support group. Support groups like this one should be established to improve the caregivers’ effectiveness to provide care to their loved one while also ensuring emotional support for themselves.

## 5. Conclusions

The dearth of research focusing on interventions for unpaid caregivers and older adults with visual impairment meant that the recruitment for this study was more challenging than anticipated. However, the results from this pilot study represent a promising program for the unpaid caregivers of older adults with visual impairments. Both quantitative and qualitative data indicated that the activities and meetings may have improved the quality of life and well-being of the participants. Future research with larger sample sizes is necessary to determine how to most effectively improve the lives of unpaid caregivers for individuals with visual impairments. In order to improve the dire consequences of the stressful experiences of caregiving for a loved one with a visual impairment, it is imperative that we develop interventions that are accessible and have the ability to increase the quality of life and life satisfaction.

## Figures and Tables

**Table 1:** Participant characteristics.

Variable	Caregiver	Care-Recipient
Age in years, median (25–75 percentile; interquartile range)	56.5 (38.5–67; 28.5)	71 (65–80; 15)
Gender, n (%)	Women 8 (66.7)	Women 7 (58.3)
	Men 4 (33.3)	Men 4 (33.3)
	Nonbinary 0 (0)	Nonbinary 1 (8.3)
Race/Ethnicity, n (%)	White 7 (58.3)	White 5 (41.7)
	African American 4 (33.3)	African American 5 (41.7)
	Latinx 1 (8.3)	Latinx 1 (8.3)
	Other 0 (0.0)	Other 1 (8.3)
Number of hours spent providing care per day, n (%)	1 to 3 h 1 (8.3)	NA
	3 to 5 h 1 (8.3)	
	5 to 8 h 1 (8.3)	
	>8 h 9 (75)	
Does the CR live with the CG, n (%)	Yes 7 (58.3)	NA
	No 5 (41.7)	
Type of visual impairment of the CR, n (%)	NA	Far sightedness/short sightedness/corrective lenses 3 (25)
		Totally blind in one or both eyes 2 (16.6)
		Macular degeneration 2 (16.6)
		Color blindness 2 (16.6)
		Retinitis Pigmentosa 1 (8.3)
		Severe blurred vision 1 (8.3)
		Other 1 (8.3)

**Table 2:** List of interventions.

List of Interventions	Facilitator(s)	Brief Description of Intervention
Tai chi	Tai chi experts; college professors	Introduced participants to various tai chi activities. It included demonstrations by a local tai chi instructor/expert, as well as college professors and research team members with expertise in tai chi as a method of health promotion.
Yoga, chair yoga, and blind yoga	Yoga instructor; college professors	Introduced participants to various forms of yoga including chair yoga and blind yoga. It included access online yoga instruction/demonstrations. Yoga activities were facilitated by a yoga instructor and college professors who are members of the research team.
Gardening	Participant/co-facilitator	Detailed information on the practice of growing and cultivating plants, particularly in an indoor-setting. This was facilitated by a participant who also acted as a co-facilitator (i.e. participant-facilitator) for some of the intervention sessions.
De-stressing techniques	Participant/co-facilitator; college professors	Detailed information and demonstration of various de-stressing techniques, including exercising, maintaining connections with others, learning to take a break, etc. The de-stressing activities were facilitated by a participant-facilitator, as well as college professors who are members of the research team.
Meditation/safari meditation	Participant/co-facilitator; college professors	Detailed information and demonstration of various meditation techniques. This included a demonstration of “safari meditation” by a college professor (and member of the research team) who had travelled to the safari in Kenya.
Music/singing	Participant/co-facilitator; college professors	Special musical presentations by a college professor and member of the research team, as well as a participant-facilitator. This also included several sing-along songs.
Dancing/ballroom dancing	College professor	Special dance presentations by a professor and member of the research team, as well as videos of ballroom dancing.
Nutrition	Nutrition expert	Presentations focusing on the benefits of proper nutrition and balanced diets.
Birding	Participant/co-facilitator	Detailed information on birding, including identifying and observing of birds as a recreational activity. This was facilitated by a participant/co-facilitator.
Numismatics	Participant/co-facilitator	Demonstration and detailed information on coin collection by a participant-facilitator.
Workshops: packing suitcases for travel, cooking, etc.	Expert in activities for visually impaired adults	Several workshops by a facilitator and expert who is blind. This includes viewing of publicly available videos that were produced by this facilitator.

## Data Availability

The data presented in this study are available on request from the corresponding author. The data are not publicly available due to privacy and ethical reasons.
